# Key Elements for Judging the Quality of a Risk Assessment

**DOI:** 10.1289/ehp.1510483

**Published:** 2016-02-05

**Authors:** Penelope A. Fenner-Crisp, Vicki L. Dellarco

**Affiliations:** 1 Independent Consultant, North Garden, Virginia, USA; 2 Independent Consultant, Silver Spring, Maryland, USA

## Abstract

**Background:**

Many reports have been published that contain recommendations for improving the quality, transparency, and usefulness of decision making for risk assessments prepared by agencies of the U.S. federal government. A substantial measure of consensus has emerged regarding the characteristics that high-quality assessments should possess.

**Objective:**

The goal was to summarize the key characteristics of a high-quality assessment as identified in the consensus-building process and to integrate them into a guide for use by decision makers, risk assessors, peer reviewers and other interested stakeholders to determine if an assessment meets the criteria for high quality.

**Discussion:**

Most of the features cited in the guide are applicable to any type of assessment, whether it encompasses one, two, or all four phases of the risk-assessment paradigm; whether it is qualitative or quantitative; and whether it is screening level or highly sophisticated and complex. Other features are tailored to specific elements of an assessment. Just as agencies at all levels of government are responsible for determining the effectiveness of their programs, so too should they determine the effectiveness of their assessments used in support of their regulatory decisions. Furthermore, if a nongovernmental entity wishes to have its assessments considered in the governmental regulatory decision-making process, then these assessments should be judged in the same rigorous manner and be held to similar standards.

**Conclusions:**

The key characteristics of a high-quality assessment can be summarized and integrated into a guide for judging whether an assessment possesses the desired features of high quality, transparency, and usefulness.

**Citation:**

Fenner-Crisp PA, Dellarco VL. 2016. Key elements for judging the quality of a risk assessment. Environ Health Perspect 124:1127–1135; http://dx.doi.org/10.1289/ehp.1510483

## Introduction

A number of U.S. federal (as well as state and local) government agencies produce risk assessments on a continuing basis. In the years since the publication of the 1983 National Research Council (NRC) report, *Risk Assessment in the Federal Government: Managing the Process* (the “Red Book,” NRC 1983), advances in risk assessment have occurred, but the need for further improvement continues to be recognized. Much attention has been focused on the risk assessment practices of the U.S. Environmental Protection Agency (U.S. EPA), although recommendations for improvement have also been directed toward other agencies. In our opinion, the problems ascribed by critics to these assessments generally do not lie in the lack of guidance on how to conduct an assessment but rather in the failure to implement internal guidance or externally generated recommendations in a consistent and transparent manner. The aim of this work was to extract from the accumulated recommendations of many expert panels a set of attributes that can serve as a guide for judging whether an assessment has incorporated consensus best practices that result in a scientifically credible, transparent, and useful product. By “best practices,” we mean that an assessment possesses scientific accountability and integrity by employing a critical, open-minded approach in selecting reliable data and models fit for their intended use and in analyzing and integrating that information. The assessment should use defined methodologies for collecting and interpreting information and for minimizing any bias that might be introduced. Its development process embraces the necessary scoping and planning before the assessment is conducted. Here, best practices ensure that transparency exists throughout to enable others to judge the scientific robustness of the conclusions and to replicate the findings and that the uncertainties associated with the assessment are described. Finally, the assessment should be readily usable and provide value to its intended audiences.

The guide presented in Appendix 1 has been designed for use by decision makers to assist in their quest to have a high-quality assessment at hand when carrying out their responsibilities and for use by authors, sponsors, risk assessors, peer reviewers, and other interested stakeholders to determine if an assessment meets the current best scientific practices. The use of the guide is intended to promote transparency and consistency with regard to the conduct and quality of assessments.

## Methods

A general consensus has been evolving over the past several years regarding the characteristics that high-quality assessments should possess. A review of a series of primarily government-funded expert panel reports was conducted to identify, assemble, and synthesize the key elements of a high-quality assessment for the purpose of creating a simple and useful quality assurance guide. These reports included those of the NRC, the Institute of Medicine (IOM), the Presidential/Congressional Commission on Risk Assessment and Risk Management, and a number of foreign governments and organizations [e.g., European Chemicals Agency (ECHA) 2011; European Food Safety Agency (EFSA) 2010, 2011, 2014a, 2014b; Health Canada 2000, 2015; IOM 2011; NRC 1983, 1994, 1996, 2007, 2009, 2011, 2013, 2014; Organisation of Economic Co-operation and Development (OECD) 2007, 2012].

## Reports Relevant to the Development of the Guide for Judging the Quality of an Assessment

The processes by which risk assessments are developed as well as their substance and content have been the subject of deliberation by many parties over the last > 30 years. The U.S. Congress, the Executive Branch, various commissions, NRC and IOM committees, affected stakeholder communities, and even the general public and individuals have all weighed in. Over time, there has been a shift in, and an expansion of, the areas of focus on the elements of the risk-assessment process. Perhaps this evolution can best be illustrated by tracking the topics addressed in a series of reports, primarily from the NRC, that began with the publication of the 1983 Red Book (NRC 1983). The U.S. Congress directed the U.S. Food and Drug Administration (FDA) to contract with the National Academy of Sciences (NAS) to conduct a study of the institutional means for risk assessment (NRC 1983). Of particular interest at that time were the interfaces and interactions between science and policy and those between risk assessment and risk management. The focus was on the potential for human health impacts of exposure to chemicals. In reality, however, the discussion is equally applicable to ecological risk assessment; to other stressors such as radiation, microbes, and products of biotechnology; and to many categories such as environmental contaminants, medical devices, drugs, tobacco, consumer products, commodity chemicals, pesticides, and food additives, constituents, and contaminants.

The Red Book committee made several recommendations for improving risk assessment through changes in procedures such as *a*) maintaining a clear distinction between the science and the other factors involved in decision making, including political considerations, economics, and technology; *b*) making a risk assessment document publicly available before finalizing regulatory decisions; *c*) subjecting the risk assessment to external expert peer review; and *d*) developing joint assessments if two or more agencies have a regulatory mandate regarding the same chemical(s) (NRC 1983). This committee also offered recommendations on improving risk assessment through the development of uniform assessment guidelines. In the committee’s view, there would be one set of guidelines that all agencies would implement. These guidelines would be detailed but flexible and would address all four phases of risk assessment: hazard identification, dose–response assessment, exposure assessment, and risk characterization. Guidelines for assessing cancer risk would be developed first, followed by those for other end points of toxicity and for exposure. Furthermore, the guidelines would be developed by a congressionally chartered board of experts who were independent of regulatory decision making.

Although the FDA funded this report, it was the U.S. EPA, under the leadership of Administrator William Ruckelshaus, that most vigorously embraced the recommendations and implemented many of them (U.S. EPA 1993). The U.S. EPA implemented all of the recommendations on procedural changes, although there are only a few examples of collaboration with other federal agencies on specific chemicals. From the mid-1980s through the 1990s, the U.S. EPA developed guidelines for cancer, mutagenicity, reproductive and developmental toxicity, neurotoxicity, chemical mixtures, and ecological effects to promote agency-wide consistency. Many other U.S. EPA–wide policies, principles, and risk-assessment guidance, databases, models, and other tools have been developed since then. Efforts to develop guidance for inter-agency use have not succeeded.

The Red Book, then, serves as the starting point for discussion, and a number of the observations and recommendations in subsequent reports can be traced back to concepts originally articulated therein. The 1994 NRC report *Science and Judgment in Risk Assessment* emphasized approaches to exposure and toxicity assessment and risk characterization as well as strategies for improving risk assessment in the areas of default options, models, data needs, uncertainty, variability, and aggregation of risk (NRC 1994). A brief discussion of priority setting laid the groundwork for future findings and recommendations related to the use of preplanning and problem formulation measures before beginning a resource-intensive assessment. An expanded discussion of problem formulation as well as of the desirability and importance of expert scientific peer review and input and comment from interested stakeholders outside of the organization preparing the assessment was addressed in the 1996 NRC report *Understanding Risk: Informing Decisions in a Democratic Society* (NRC 1996). The value of problem formulation and planning and scoping before conducting an assessment was also emphasized in the findings and recommendations of the Presidential/Congressional Commission on Risk Assessment and Risk Management, which stated that “The level of detail considered in a risk assessment and included in a risk characterization should be commensurate with the problem’s importance, expected health or environmental impact, expected economic or social impact, urgency, and level of controversy, as well as with the expected impact and cost of protective measures” (Presidential/Congressional Commission on Risk Assessment and Risk Management 1997).

The 2009 NRC report *Science and Decisions: Advancing Risk Assessment* revisited topics such as uncertainty and variability, defaults, and cumulative risk assessment, along with new issues related to dose–response assessment (NRC 2009). The importance of the design of risk assessment processes to improve their utility for decision making was also addressed. This committee observed that “the selection of appropriate elements of process and the specification of required elements of the final product constitute a complex design challenge” (NRC 2009). They viewed the incorporation of “fairness, transparency and efficiency” in both the process and the resulting assessments as critical elements to assure the quality and usefulness of the assessments to both decision makers and other stakeholders. “Objectivity” and “balance” are also essential characteristics of high-quality products. To provide some structure to the risk-assessment process, this committee presented its vision of a framework for risk-based decision making that, in its view, would make the best use of a risk assessment. Similar in structure and content to the framework schematics included in a number of the U.S. EPA’s guidance documents (e.g., U.S. EPA 1992, 2003, 2006, 2014a), this framework describes three general components: the first includes problem formulation and scoping, the second reflects planning as well as the technical components of the risk assessment itself, and the third focuses on other factors (e.g., legal, technological, economic) that must be considered to reach and communicate management decisions. Risk assessment frameworks developed by other governments and organizations also emphasize the importance of problem formulation as a first step [e.g., Health Canada 2000; World Health Organization International Programme on Chemical Safety (WHO IPCS) 2010; EFSA 2013].

Continuing dissatisfaction with the perceived lack of adequate documentation and transparency in the selection and interpretation of data and with the application of science policy guidance, as well as the perception that many risk assessments do not reflect the incorporation of the best available science, has focused attention on the concept of systematic review (NRC 2014). Systematic review is defined by the IOM as “a scientific investigation that focuses on a specific question and uses explicit, prespecified scientific methods to identify, select, assess, and summarize the findings of similar but separate studies” (IOM 2011). We contend that implementation of such an approach would serve to ameliorate at least some of the concerns.

Systematic review has been used for several decades in the fields of medicine, education, and agriculture. If done properly, it can improve the credibility of the assessment. Although the early focus of systematic review was on clinical medicine and health care, beginning with the development of the Cochrane Collaboration (Higgins and Green 2011), the importance of a more formalized procedure is now recognized as important for human health and ecological assessments of chemical and environmental exposures [e.g., National Toxicology Program (NTP) 2015; Texas Commission on Environmental Quality (TCEQ) 2014; U.S. EPA 2013; Woodruff and Sutton 2014], food and feed safety (e.g., EFSA 2010; FDA 2009), and guideline development (WHO 2012). In its report *Review of the Environmental Protection Agency’s Draft IRIS Assessment of Formaldehyde*, the NRC argued strongly for the implementation of a systematic review process in the development of Integrated Risk Information System (IRIS) assessments (NRC 2011). While acknowledging that the U.S. EPA had made some progress on incorporating elements of systematic review into its IRIS document development process since 2011, the NRC Committee to Review the IRIS Process pressed forward with additional comments and recommendations on problem formulation and protocol development, evidence identification, evaluation, and integration for hazard characterization as well as on methodological issues related to dose–response assessment and the derivation of toxicity values (NRC 2014). This committee concluded that the general approaches and concepts underlying systematic reviews for evidence-based medicine embodied in the standards established by IOM should generally be relevant to the review of animal, epidemiologic and mechanistic studies in the IRIS hazard characterization process. One might argue that it would also be relevant to assessments prepared by other parties. Systematic review should also be presumed to be applicable and useful in assessments that include exposure assessment and risk characterization.

Systematic review has already been embraced by the NTP (2015) and the EFSA (2010, 2011) and is beginning to be implemented by several U.S. EPA offices (U.S. EPA 2012, 2013). Although some efforts focus on hazard identification alone (NTP 2015) or on hazard identification and dose–response assessment (EFSA 2010; U.S. EPA 2013), others cover exposure assessment and risk characterization as well (EFSA 2011; U.S. EPA 2012).

The later steps of the systematic review process—interpreting results and drawing conclusions—flow into the assessment itself. The assessment is conducted against the backdrop of a predetermined scope that defines the linkages between stressors (chemical or other) and adverse human health or ecological effects, including identifying the stressor(s), exposure pathway(s), exposed life stage(s) and population(s), and toxicity end point(s) of concern that will be addressed in the assessment (U.S. EPA 1992, 1998, 2014a). The result of this effort is a complete assessment, comprising several components, which may or may not be issued at the same time. These components might be staged, beginning with a problem formulation/planning and scoping product issued first, which might be subjected to peer review and public comment. A second product, the systematic review, might also be subjected to peer review and public comment. Finally, the assessment itself, reflecting an objective, scientific analysis of the key data with a transparent identification of relevant science policy choices (e.g., application of defaults, selection of dose–response models, use of uncertainty factors) might be subjected to peer review and, perhaps, to public comment.

## Discussion of the Guide for Judging the Quality of an Assessment

The ultimate purpose of the guide is to provide guidance for evaluating the quality of an assessment. We envision the guide to be used both as a self-assessment tool by the author(s) of an assessment and as a mechanism for judging the quality of an assessment prepared by another party. For the purposes of discussion and simplification, we are viewing transparency and usefulness as desirable characteristics of quality and are folding them into the single term of “quality.” How, then, should the quality of an assessment be judged? And which assessments should be subjected to such an analysis?

Although the criticisms have been directed most frequently at the perceived weaknesses and inadequacies of U.S. EPA assessments, those of other federal agencies have also received attention. However, we would argue that the standards of performance demanded of the U.S. EPA and other federal agencies should also be demanded of state and local government agencies, communities, regulated industry, public interest groups, academics, and any other parties that conduct or fund risk assessments and related research on their own behalf. Their products should also be subjected to external expert peer review, public comment, and a quality analysis. In other words, it should not only be the U.S. EPA and other federal agencies that are obligated to upgrade their assessment processes and practices and prove their credibility; it is incumbent upon the government’s involved stakeholders to do the same, especially the regulated community. In particular, if a nongovernmental entity wishes to have its assessments be considered in the governmental regulatory decision-making process, then its products should be judged in the same rigorous manner as that expected of the government.

Transparency, effectiveness, efficiency, and scientific integrity are all essential traits that are captured in the Guide. These features are applicable to any type of assessment, whether it encompasses one, two, or all four phases of the risk-assessment paradigm, whether qualitative or quantitative, screening level or highly sophisticated and complex. These characteristics apply to both traditional approaches and to the newer 21st century or “next-generation” approaches, as described, for example, by the NRC (2007, 2012) and the U.S. EPA (2014b).

Organizations, government and otherwise, should rightfully be held accountable to their respective constituencies. A carefully crafted set of performance measures can serve as a credible tool for determining the level of success in meeting performance expectations. In the case of the U.S. federal government, each agency of the Executive branch is required to submit annual performance reports to the Office of Management and Budget (OMB) (OMB 2016). These reports represent data-driven reviews of the strategic objectives established in their respective Strategic Plans and include an articulation of achievements made toward meeting program objectives along with identification of areas where improvement may be needed. Among the purposes served by these reports are informing long-term strategic decision making; facilitating identification and adoption of opportunities for improvement; identifying areas where additional evaluation, other studies, or analyses of data are needed; identifying where additional skills or other capacity are needed; strengthening collaboration on crosscutting issues; and improving transparency. Similar activities performed in the private and nonprofit sectors are viewed as reflective of good management practices. Progress is measured against a set of predetermined performance criteria. We see the value of employing the guide as an application of the same concepts to an evaluation procedure for judging the quality of assessments. Given the growing consensus among the many interested parties of what characteristics a high-value assessment should possess, it is useful to have a guide, available as a single document, to provide direction for authors, decision makers, reviewers, readers, and other users when they are judging the quality of such an assessment.

What key elements might one document include in order to determine whether an assessment meets the criteria for a high-quality product? The guide contains a series of points focused on good scientific practices, as gleaned from the expert panel reports, to be used in developing credible and transparent assessments. We acknowledge that the measures presented may not be all-inclusive, but we believe that they capture the key considerations necessary for a high-quality assessment. In the following sections, we highlight a few overarching themes around the points captured in the guide.

## Designing with Focus

The guide begins with the foremost characteristic of a high-quality assessment: fit-for-purpose. That is, the assessment clearly addresses the problem(s) and questions at hand and considers the options or boundaries for which decisions need to be made. Before an assessment is initiated, problem formulation, planning, and scoping must occur. These are crucial steps for producing an effective and efficient assessment. A number of points must be addressed, such as the overall purpose and general scope of the assessment, the assessment products needed to inform decision making, the resources required, who the authors of the risk assessment will be and their respective roles, the time table, in addition to other considerations. (U.S. EPA 2014a).

Good problem definition needs to address the issues and concerns of the key participants and stakeholders. Critical to public confidence and a successful product is an open process that allows early and continuing dialogue with the stakeholder community. Stakeholders can serve a valuable role in identifying issues, data, and alternative approaches to conducting an assessment.

It is worthwhile to note that organizations are usually faced with finite resources and time to conduct their assessments; thus, not only are there scientific drivers that demand improved quality but also the realization that resources must be used efficiently. The extent of documentation needs to be balanced by resources and priorities, particularly when the timeliness of the response is critical (Dellarco et al. 2010; Health Canada 2000). The mere presence of a substance in the environment does not necessarily mean that it poses a threat to human health or to the environment; thus, an approach that considers exposure early in the process can better focus resources on those stressors that pose exposure scenarios of concern. Some NRC reports (NRC 1996, 2009) and other publications (Pastoor et al. 2014) have also noted that problem formulation must include an early consideration of the relevant exposure scenarios/pathways along with potential options for managing or mitigating the exposures. Only then will the assessment efficiently and effectively serve the needs of the user. In all cases, transparency is key; the selected approaches should be well described in the problem formulation document.

## Selecting Data and Evaluating Reliability

Once the problem-formulation phase is completed, a systematic review process should follow. An important aspect of this process is the definition and documentation of the search and review procedures employed to ensure both transparency and that the results can be replicated. The literature search and the procedures for data collection and evaluation will shape the scientific basis of the assessment and thus need to be guided by the questions, goals, and methodologies identified in the problem-formulation phase. It should be emphasized that this is an iterative process.

The systematic review process is designed to reveal and minimize bias. Although it is difficult to eliminate bias completely, if all documents (including the documented search for studies) have been made publicly available, and the reasoning behind choices has been made clear, then, at the least, bias can be identified, addressed, and minimized. Furthermore, having an assessment team of multidisciplinary scientists can ensure a range of perspectives that permits alternative interpretations to be considered along with the evidence that supports or refutes these alternatives.

Only well-documented epidemiologic, toxicity, and exposure studies based on reliable data should be used, particularly in an assessment that could have a significant impact on the identification and selection of decision options. Studies that are poorly documented or those with questionable study design and reproducibility should be identified as such and should not be used. These judgments would become clear in the course of conducting a scientifically rigorous and transparent systematic review. The evaluation of a study’s quality is essential to the weight-of-evidence (WoE) process. In 2001, the OMB issued guidelines that ‘‘provide policy and procedural guidance to federal agencies for ensuring and maximizing the quality, objectivity, utility, and integrity of information” (OMB 2001). Each federal agency was required to issue implementing guidelines (e.g., DHHS 2002; U.S. EPA 2002). Foreign governments and international organizations have developed their own quality guidelines (ECHA 2011; Health Canada 2015; OECD 2007, 2012).

## Combining Evidence

Once the candidate studies have been assembled and evaluated for quality, the relevant credible body of data is subjected to a WoE/evidence integration exercise in order to characterize the extent to which the hypotheses put forward are or are not supported. This aspect of risk assessment has been the most difficult to execute well. Authors may assert that they have weighed and integrated all of the information in a constructive manner, but their interpretive approach and how it was applied is often unclear or lacking in documentation (NRC 2011). If the integration process is based upon well-defined criteria that ensure structure and rigor, it can be of important value and scientific use. Rhomberg et al. (2013) reviewed nearly 50 frameworks to evaluate best practices for WoE analyses for determining causation of chemical risks, concluding that the review, along with its companion workshop deliberations, provided “actionable best practices recommendations that can be put to immediate use in designing and conducting systematic reviews/WoE determinations for chemical hazards/risks.” In separate studies, Rhomberg and colleagues developed a hypothesis-based approach for synthesizing dissimilar and complex data sets to more successfully support a true WoE output (Rhomberg et al. 2010; Rhomberg 2015). Other hypothesis-based WoE approaches (using Bradford Hill–like considerations) have been developed that also promote systematic evaluation and integration of data: in this case, to characterize toxic modes of action and their relevance to human targets. The mode-of-action/human relevance framework developed by WHO/IPCS and the International Life Sciences Institute focuses on human health (Boobis et al. 2006, 2008; Meek et al. 2003; Seed et al. 2005; Sonich-Mullin et al. 2001). Many examples exist of the application of this framework and the incorporation of its results into the decision-making process by regulatory authorities, both domestic [California Environmental Protection Agency (CalEPA) 2013; U.S. EPA 2005, 2010] and international [EFSA 2014a, 2014b; Food and Agriculture Organization of the United Nations/World Health Organization (FAO/WHO) 2012, 2013]. The WHO/IPCS framework has evolved to incorporate the use of quantitative dose–response and temporal relationships for key events within a mode-of-action to reduce uncertainty and to better inform quantitative risk assessment (Julien et al. 2009; Simon et al. 2014) along with other refinements as experience with its application accrues (Meek et al. 2014a, 2014b). In its development of a library of adverse outcome pathways (AOPs), the OECD is coordinating its activities with WHO/IPCS and has incorporated the WoE approach of the WHO/IPCS framework into its guidance and template to assess the evidence in support of an AOP (OECD 2013). Conceptually similar to mode-of-action, an AOP is defined as “an analytical construct that describes a sequential chain of causally linked events at different levels of biological organisation that lead to an adverse [human] health or ecotoxicological effect. AOPs are the central element of a toxicological knowledge framework being built to support chemical risk assessment based on mechanistic reasoning” (OECD 2013). At present, AOPs are being developed to address the common goal of identifying faster, more reliable interpretable methods [(e.g., *in vitro* screens, strengthening read-across methods, and quantitative structure-activity relationship models (QSARs)]. Understanding of AOPs, in turn, informs mode-of-action analyses for specific chemicals or groups of chemicals.

## Ensuring Transparency and Clarity

It is essential that an assessment be both transparent (i.e., possess openness in procedural process and scientific aspects) and easily understood (i.e., possess clarity). Scientific assessments can be hard to follow, even for a technical audience. However, it should never be assumed that difficulties in understanding an assessment are simply a result of the complexity of the scientific data used, the type of analysis, and the concepts applied. Rather, they can also be the result of deficiencies in communication. Clarity is an important feature of any quality assessment. Mere presentation of information is insufficient for an assessment intended to support decision making. The points at which choices are made in the selection of data, the use of defaults and assumptions, the consideration of alternative methods, and so forth, and the reasoning underlying these choices need to be clearly captured for users and other readers. The inclusion of an executive summary written for both technical and nontechnical audiences, in addition to summary tables and figures, can facilitate the reader’s comprehension of the assessment. Clarity in the assessment is particularly important in situations involving difficult determinations about data interpretation or conflicting views on plausible alternative methods.

Uncertainty is a scientific reality that cannot be totally eliminated, and, therefore, must be acknowledged and explained, including its impact on the risk conclusions and estimates. It is essential that all conclusions and risk estimates (including alternatives) be described explicitly in the context of certainties and uncertainties. Uncertainties that are identified should be based on empirical knowledge rather than on speculative “unknown negative effects.” In general, the nature of any uncertainty raised should be at least addressed qualitatively, if not quantitatively. It is important for an assessment to identify ways in which uncertainty could be reduced. Progress has been made in the tools to characterize uncertainty (particularly for exposure), and guidance is available (e.g., WHO IPCS 1999, 2009; U.S. EPA 2000). Nonetheless, there remains a need to strengthen this area of assessment, as noted in the NRC report *Environmental Decisions in the Face of Uncertainty* (NRC 2013).

### Objectivity and Reasonableness

There are a number of potential sources of bias that can occur in the various phases of the assessment process (e.g., study design, data selection, data interpretation, choice of defaults, models, methods). One area of bias concerns author bias (i.e., bias on the part of those conducting the assessment). It should be acknowledged here that scientists/risk assessors have biases, beliefs, and opinions; however, it is critical that advocacy positions not infiltrate and influence an assessment. A credible assessment that embodies best scientific practices is based upon empirical evidence and ensures scientific objectivity.

While remaining mindful of the goal to protect human health and the environment at reasonable cost, a truly useful assessment should reflect the common-sense application of assumptions and policies and avoid providing mischaracterizations of hazard or unrealistic estimations of exposure and risk (in either direction). A reality check is important to ensure that conservative assumptions have not over-accumulated in the assessment. Scientifically sound characterization of hazard and realistic estimates of exposure and risk lend credibility to, and improve confidence in, the assessment. Related to this point is the consideration of alternative conclusions or risk estimates that have reasonable supporting evidence. Presenting feasible alternatives provides more information for consideration in decision making and lends more credibility to the assessment; this is an important responsibility of the authors of an assessment. The regulatory process demands consideration of the full range of possibilities. Robust characterization of all of the supportable alternatives is critical to the users of the assessments, especially decision makers, and it is an essential characteristic of a credible assessment.

### Peer Reviewing the Science

Scientific peer participation and peer review are key elements of the assessment process. They play a critical role in ensuring the credibility and integrity of the scientific information generated, evaluated, and communicated by the authors. Peer participation includes involvement in the development of an assessment. Independent peer review of a draft assessment can be a reliable judge of the usefulness, quality and relevance of the assessment; it can also evaluate the scientific objectivity and the consideration of alternative interpretations and methods.

Agencies of the federal government are expected to develop and execute a formal peer review process; this is mandated, in part, by the OMB. Its Peer Review Bulletin established “government-wide guidance aimed at enhancing the practice of peer review of government science documents” (OMB 2004). The Peer Review Bulletin also required that each federal agency develop and publish a process for conducting peer reviews that is transparent, incorporating the Bulletin-established minimum standards for when and what type of peer review is required and appropriate for a given circumstance. In addition, if a peer review is being conducted as a component of activities covered by the Federal Advisory Committee Act (FACA), additional steps may need to be incorporated into the process (Federal Advisory Committee Act 1972). FACA provisions also apply to committees convened by the National Academy of Sciences (NAS 2003).

A number of state governments have also developed policies and guidance with regard to peer review. Some practices are mandated under state law (e.g., Bowes 2006). Some state governments convene standing advisory panels/committees, which may conduct peer review of their government’s assessments or develop their own assessments. These panels generally convene in a public forum and/or provide opportunities for interested stakeholders to provide written review and comment before finalizing their recommendations to their respective state. The peer review policies and practices that may exist in local governments are less clear. Nonetheless, there is great demand that all levels of government function in an open and transparent manner and provide for stakeholder input at various points in the decision-making process.

However, the regulated community, public interest groups, academics, and other parties that prepare or fund assessments or related research on their own behalf have no obligation to perform the same type of open and collaborative peer review. Rarely, their assessments are peer-reviewed by a panel convened by a third party. However, these panels generally do not perform their evaluations in a public setting, do not solicit public comment, and their membership and deliberations may or may not be made public after the fact.

Authors/sponsors may opt for publication of their assessment or research in a peer-reviewed scientific journal. Doing so serves two purposes: *a*) the information is peer-reviewed, presumably by qualified experts, and *b*) the final product can be widely disseminated to the interested audience. However, the peer-review process for journals is generally not very transparent. Although a reader may have access to the final publication, who performed the peer review or how the authors responded to any comments received is seldom revealed. In many cases, there is not access to sufficient information for the reader to attempt a replication of the assessment or research study, although some journals now allow authors to provide supplementary information with their manuscripts. Further improvements in the journal publishing arena may arise in light of the issuance of the Principles and Guidelines for Reporting Preclinical Research, which were agreed upon in a gathering of more than 30 major journal editors, representatives from funding agencies, and scientific leaders that was convened by NIH and the journals *Nature* and *Science* (NIH 2015b). Application of these principles and guidelines will serve to guide and enhance the development of a harmonized, systematic review process. Of particular value are the recommendations made in the areas of rigorous statistical analysis, transparency in reporting, and data and material sharing. The effort with regard to data sharing has already begun within the federal government at the direction of the Office of Science Technology and Policy. A memo issued in February 2013 directs “each Federal agency with over $100 million in annual conduct of research and development expenditures to develop a plan to support increased public access to the results of research funded by the Federal Government. This includes any results published in peer-reviewed scholarly publications that are based on research that directly arises from Federal funds” (OSTP 2013). To date, ≥ 14 agencies have finalized their plans (e.g., FDA 2015; NIH 2015a). The U.S. EPA’s final public access plan has yet to be issued.

## Applying the Guide

The guide is intended to be both a self-assessment tool and a tool to enable others to judge the robustness of an assessment. We would expect that any individual who employs the guide to judge the quality of an assessment would do so based upon an understanding of the policies and practices of the authoring organization and informed by his/her experience, knowledge, and perspectives, while remaining vigilant to minimize any personal bias. There may well be situations in which an individual possesses or is perceived to possess a conflict of interest and, thus, may not be the best candidate to perform the quality review. The guide could also serve as an especially valuable companion piece to the charge that peer reviewers receive from a sponsoring organization when they are asked to evaluate an assessment. Although an assessment may not “tick every box” for all of the elements in the guide, this does not necessarily mean that the assessment is not of high quality. Whether or not an assessment possesses all or just some of the attributes identified in the guide will depend upon the nature of the assessment (e.g., screening vs. in-depth or encompassing only one or more elements of the risk-assessment paradigm). This is determined by the scope as developed during the problem-formulation phase. In any case, even if some attributes are not present, having this noted in the application of the guide allows for enhanced transparency. If the guide application results are released along with the assessment, readers can easily see which attributes were incorporated and which were not. This level of transparency will be useful to decision makers, peer reviewers, and other interested parties.

## Conclusions

A review of > 30 years’ worth of NAS/NRC/IOM and other federal government–funded expert panel reports coupled with government agency science and policy guidance developed in response to these reports reveals the degree of consensus around the key elements that a high-quality and useful assessment should possess. These key elements have been used to develop a guide by which decision makers can determine if they have the highest quality information available to carry out their responsibilities and by which authors, sponsors, risk assessors, peer reviewers, and other stakeholders can determine if an assessment reflects current best scientific practices.

Over the years, many reports have been published concerning the quality, transparency, and usefulness for decision making of human health and ecological assessments prepared by agencies of the U.S. federal government. Recommendations for improvement have been offered on every aspect of the design, content, execution and role of risk assessments in the decision-making process. From the plethora of observations and recommendations, a consensus is evolving with regard to the characteristics that an assessment should possess to be deemed of high quality. Each federal agency is mandated to conduct data-driven performance reviews of their priorities to gauge their progress toward achieving their stated goals (OMB 2016). Successfully functioning state and local governments and organizations in the private, non-profit, and academic sectors also often incorporate such reviews as a matter of good management practice. Capitalizing on this precedent, we have created a guide that provides the author, decision maker, risk assessor, peer reviewer, or other interested stakeholder a means for determining whether a particular assessment meets the criteria for excellence. We envision this guide to be especially useful as a self-evaluation tool and as a companion piece to consult during the problem-formulation process. It can also serve as a complementary component of the charge received by peer reviewers from a sponsoring organization when they are asked to evaluate an assessment. We believe it would be prudent to use the guide (or a modified version thereof that is appropriately tailored for its scope and for a technical peer review) as a gauge for determining the quality of an assessment in advance of circulating the assessment to a larger audience for review and comment and before any regulatory decisions are made.

The guide, constituting a series of statements/measures, covers many aspects of problem formulation, systematic review of the literature, hazard identification/characterization, dose–response assessment/characterization, risk characterization, and peer review. The statements capture key aspects of the essential traits of an assessment around which consensus has emerged. When the totality of applicability to the statements is viewed holistically, as in a WoE evaluation, one should be able to easily gauge the level of accomplishment in meeting the objectives of creating a good assessment. Simply put, the more often a characteristic of the guide can be ascribed to the assessment being judged, the more likely it will be seen as meeting its performance expectations.

## Appendix 1: Guide for Judging the Quality of an Assessment

### Problem Formulation/Scoping and Planning

Possesses effective focus on identified risk management questions/options, and results in the depth/extent of the assessment are commensurate with the nature and significance of the decisions to be made.
Issues pertaining to relevant exposure scenarios, data collection and evaluation, methods of assessment including mode of action (MoA) analysis, ongoing research, etc. are identified.
Presents evidence that a dialogue occurred with scientific and stakeholder communities that afforded a reasonable opportunity for their input on key issues before (and while) preparing the assessment.

### Systematic Review of the Literature

The search strategy (including predefined study inclusion/exclusion criteria, literature sources, search terms) used to identify relevant literature (both negative and positive studies) is well documented and is available to the public. Any restrictions placed on the literature search or data access are noted and explained.
Evidence is presented that outside parties were given a reasonable opportunity to provide relevant studies that were not identified in the authors’ literature review and to comment on the quality of the studies selected for inclusion.
Sufficient data for the critical studies and the models used in the assessment are available to interested external parties to enable them to replicate/verify the assessment outcomes and to judge the scientific credibility of the data/models.

### Hazard Assessment/Characterization

The quality (i.e., reliability, validity of the method/study design), relevance, and utility of the results of each study are evaluated using an objective approach that employed predefined criteria.
An *a priori* established weight-of-evidence approach addressing causal relationships is applied in a systematic manner to integrate and effectively use all relevant information and to weigh the lines of relevant evidence. Both positive and negative studies are weighed objectively. Judgments and choices made are transparent.
A robust discussion takes place about key lines of evidence and inherent uncertainties, alternative interpretations, other issues that may have prompted debate, and how these issues are addressed.
Questions about whether a response was adverse are identified, explained, and addressed.
Confounding factors and the extent to which other stressors might cause or affect adverse effects (e.g., potential antagonistic/synergistic effects) with the subject chemical are considered and addressed.
Depending on available information and to the extent possible, MoA data are taken into account, evaluated in a systematic manner using predefined criteria, and are fully incorporated into the assessment of the key end points, dose–response relationships, human relevance, and life stage impacts.
The MoA analysis includes a consideration of category analogs as a complement to stressor-specific data, and existing knowledge is effectively leveraged on previously established MoAs similar to those of the substance of interest.
A discussion takes place about whether the key events within the MoA would progress to an adverse effect relative to concentration/dose and anticipated human exposure (duration/magnitude/route) and to life stage.
Depending upon the purpose of the assessment, if the stressor produces an effect through an MoA by which other stressors have been shown to operate, the need for a cumulative assessment is identified.

### Dose–Response Assessment/Characterization

The end points used in the dose–response assessment are those that are most strongly causally associated with adverse responses, are biologically plausible in humans, and are derived from studies that meet standards of acceptable quality.
The nature of responses (e.g., biochemical, morphological, physiological, or functional change; severity of the effects; reversibility) and their dose responses [e.g., steepness or shallowness of dose–response curve, dose spacing between the no-observed-adverse-effect level (NOAEL) and the lowest-observed-adverse-effect level (LOAEL)] are described.
The dose responses are plotted for more than one end point of concern, and a distribution of hazard values or points of departure (PODs) is provided for all relevant end points. The selection of the hazard values is well justified and supported by the overall database.
Consistent with the level of complexity needed, and if the data support modeling, multiple approaches are carried forward in the analysis, and a justification is provided for model selection.
Default assumptions are identified, the rationale for each is explained, and their impact on the assessment’s conclusions is described.
What is known about endogenous production and naturally occurring background levels of the subject chemical is considered, and if appropriate, is incorporated into the analysis.
Dose–response relationships are assessed for critical windows of exposure/susceptibility.
Consistent with the level of complexity needed, and if available, suitable toxicokinetic and toxicodynamic data are used to derive refined dose–response estimates.
Consistent with the level of complexity needed, and if available, quantitative dose responses (and timing) of key events within an MoA are incorporated into the model.

### Exposure Assessment/Characterization

An understanding of the chemical’s physiochemical properties, distribution, and fate in the environment are reflected in the assessment.
Chemical breakdown products are considered.
Relevant populations are identified and assessed including demographic factors (e.g., life stage/age, sex, ethnicity), geography, and human activity patterns that would make a given group more vulnerable to exposure than others.
Depending on the purpose of the assessment, occupation-related activities that bring workers into contact with the chemical are identified and assessed.
Intended uses, significant sources, and scenarios of exposure (e.g., routes, frequency, and durations) are identified and evaluated.
The sources of information used to derive the exposure estimates are described (e.g., generic data, generic data with chemical-specific attributes, chemical-specific exposure monitoring data, or internal dose data), and inherent uncertainties are identified.
If models are used to estimate exposure, their strengths and limitations are clearly described, and sufficient information is available to enable others to replicate and verify the models.
If only conservative, worst-case estimates of exposure are generated, the rationale for the approach is provided.
If the analysis is deterministic and did not employ a probabilistic approach, a justification is provided.
Sufficient, reliable data are used in lieu of default assumptions.
Resulting certainties/uncertainties and default assumptions used are identified, and a sensitivity analysis is conducted to evaluate the impact on conclusions.
If only minimal information was available to assess exposure (e.g., physicochemical properties, molecular weight, vapor pressure solubility in fat and water), additional information needs are identified.

### Risk Characterization (In this phase, the findings on hazard, dose response, and exposure characterizations are summarized, and an integrative analysis is developed. The elements herein should also have been addressed under the other three phases of risk assessment.)

It is written for both technical and non-technical audiences and is clear and understandable in describing the purpose/objectives, scope, and main findings.
Consistent with the scope and context, all potential hazards/risks are presented for the populations and exposure scenarios of interest.
It reflects an appropriate matching of hazard and exposure data characterized by life stage and exposure scenario. If not, the assumptions used and their impact are described.
It is consistent with data that meet the relevance and quality criteria, and it reflects a minimization of bias on study design, data selection and interpretation, choice of models, and conclusions.
Scientific facts and science policy choices are clearly distinguished. Confidence in conclusions/risk values is placed clearly in the context of certainties and uncertainties, and the reasoning for the use of and the impact of defaults on conclusions are explained.
If a quantitative uncertainty analysis is provided, it is probabilistic, and the data, methods, and models used are described to allow for independent reanalysis. If not, a justification for not doing a quantitative analysis is provided.
Variability in effect or response across relevant populations(s) is discussed, and significant uncertainties are noted.
Alternative judgments, hypotheses, and models are presented along with support for these alternatives. If the assessment includes only a worst-case scenario, an explanation is provided.
Significant data needs are clearly identified. There is a discussion of the potential impact such data might have on the assessment (i.e., the value of the information).
If appropriate, risk–risk comparisons are included to provide context for the decision maker.

### Peer Review

A documented process for peer review consistent with the agency’s/sponsor’s guidance/policies and its extent and nature matches the purpose/scope and potential impact of the assessment. This process is conducted before the assessment is finalized.
Conflicts of interest and bias are identified and addressed.
All draft materials are available to public commenters and peer reviewers at a similar time, and adequate time is allowed for public comment.
Peer reviewers receive the public comments in advance for adequate consideration before the peer review meeting is conducted.
There is a reasonable opportunity for public comments to be presented at the public peer review meeting, and there is an opportunity for peer reviewers to engage with public commenters on the key technical issues they put forward.
If peer reviewers do not reach consensus, a minority opinion/report is provided.
Public and peer review comments are objectively and appropriately addressed by the party who authored the assessment.
